# Sagittal Alignment and Segmental Mobility After Cervical Intradural Extramedullary Tumor Surgery: A Comparative Analysis of Unilateral Hemilaminectomy and Laminotomy with Laminoplasty

**DOI:** 10.3390/jcm15072672

**Published:** 2026-04-01

**Authors:** Jae Min Kim, Yong Eun Cho, Keun Su Kim, Hyun Jun Jang, Bong Ju Moon, Jun Jae Shin

**Affiliations:** 1 Department of Neurosurgery, Daejeon Woori Hospital, Daejeon 35262, Republic of Korea; daejibe@naver.com; 2 Department of Neurosurgery, Spine and Spinal Cord Institute, Gangnam Severance Hospital, Yonsei University College of Medicine, 211 Eonju-ro, Seoul 06273, Republic of Korea; 3 Department of Neurosurgery, Leon Wiltse Memorial Hospital, Suwon 16480, Republic of Korea; 4 Department of Neurosurgery, Yongin Severance Hospital, Yonsei University College of Medicine 363, Dongbaekjukjeon-daero, Yongin 16995, Republic of Korea

**Keywords:** cervical spine, intradural extramedullary tumor, hemilaminectomy, laminotomy, facet preservation, segmental range of motion

## Abstract

**Objectives**: In this retrospective comparative cohort study, we aimed to compare surgical efficiency, radiographic facet integrity, and postoperative alignment and mobility between unilateral hemilaminectomy (UL) and laminotomy with laminoplasty (LP) for cervical intradural extramedullary (IDEM) tumors. **Methods**: Thirty-eight patients (UL: 20; LP: 18) were retrospectively reviewed. Operative variables, tumor characteristics, extent of resection, radiographic facet joint violation (graded 1–4), and sagittal alignment parameters, including global and segmental range of motion (ROM), were evaluated at 1 year postoperatively. Propensity score matching was additionally performed to minimize potential baseline imbalance between groups. **Results**: The UL group had significantly shorter operative time (178.05 ± 61.89 vs. 276.06 ± 121.76 min, *p* = 0.003) and lower intraoperative blood loss (*p* < 0.001) than the LP group. Radiographic facet joint violation (Grade ≥ 2) occurred more frequently in the UL group (25.0% vs. 0%, *p* = 0.048) but was not associated with postoperative sagittal alignment changes or radiographic instability. Global cervical alignment remained in both groups, but the LP group showed a significantly greater reduction in segmental ROM at 1 year (−6.42 ± 8.29° vs. 0.06 ± 7.72°, *p* = 0.017). These findings were consistent in the propensity score–matched cohort. **Conclusions**: UL provides favorable operative efficiency and better preservation of segmental cervical mobility than LP, while maintaining comparable clinical and radiographic outcomes. Although radiographic facet joint violation was more frequent in the UL group, postoperative spinal stability was not compromised in this cohort. UL may serve as a safe and motion-preserving alternative in selected patients with cervical IDEM tumors.

## 1. Introduction

Intradural extramedullary (IDEM) tumors constitute approximately two-thirds of all primary spinal neoplasms [1]. As the majority of these lesions are benign, surgical excision is the standard of care, with gross total resection (GTR) providing excellent long-term prognosis and low recurrence rates [2,3]. Traditionally, total laminectomy—an invasive procedure involving the removal of most or all of the posterior vertebral arch—has been performed to achieve adequate neural decompression; however, this approach may compromise spinal stability and necessitate additional fusion procedures.

To reduce postoperative instability while maintaining a sufficient surgical corridor, laminotomy with laminoplasty (LP), which preserves a portion of the lamina, has been adopted as an alternative technique. Nevertheless, both total laminectomy and LP typically require bilateral detachment of the paraspinal musculature and extensive disruption of the posterior bony elements. The resulting violation of the posterior tension band is a well-recognized risk factor for post-laminectomy kyphosis [4], which may lead to chronic axial pain and secondary neurological deterioration [5]. To mitigate these sequelae, unilateral hemilaminectomy (UL) has been utilized as a minimally invasive alternative that preserves spinous processes and contralateral musculoskeletal structures [6,7,8].

Biomechanically, the cervical spine exhibits the greatest overall mobility within the vertebral column, particularly with respect to rotation and combined movements, whereas the lumbar spine is most mobile in flexion and extension, and the thoracic spine remains comparatively rigid in the sagittal plane. Given this unique mobility profile of the cervical spine [9], preservation of the paraspinal musculature and facet joints may be especially important for maintaining postoperative segmental motion and alignment. Accordingly, quantifying the biomechanical and clinical benefits of preserving the posterior structures in the cervical region is of particular relevance.

Previous studies have demonstrated the safety and efficacy of UL, especially in preserving posterior elements and reducing postoperative deformity. However, a significant research gap remains as most existing reports have analyzed heterogeneous cohorts encompassing cervical, thoracic, and lumbar segments [6,10,11]. Given the unique biomechanical profile and high mobility of the cervical spine, findings from other spinal regions cannot be directly extrapolated. Furthermore, previous studies have focused primarily on static sagittal alignment, and the specific impact of unilateral versus bilateral posterior muscle detachment on dynamic cervical mobility remains poorly understood.

This retrospective study compares the clinical and radiographic outcomes of UL and LP in patients undergoing surgical resection of cervical IDEM tumors. We evaluated the relationships among cervical alignment parameters, segmental mobility, and neurological recovery, while ensuring equivalent tumor resection efficacy between the two approaches. Additionally, we investigated whether postoperative changes in cervical alignment and mobility parameters could predict neurological recovery following surgical treatment of cervical IDEM tumors.

## 2. Materials and Methods

### 2.1. Patient Selection

This retrospective cohort study included patients who underwent surgical resection of cervical IDEM tumors at our institution between January 2018 and December 2024. Patients were eligible if they: (1) had a pathologically confirmed IDEM tumor; (2) underwent either UL or bilateral LP group; and (3) completed at least one year of postoperative radiographic evaluations, including neutral and flexion–extension lateral cervical radiographs.

To minimize confounding biomechanical effects, patients with dumbbell-shaped tumors including an extradural component, intramedullary tumors, or those who underwent total laminectomy without screw or fusion procedures were excluded. All surgeries for simple IDEM tumors meeting the inclusion criteria were performed by two senior surgeons. One surgeon (Y.E.C.) performed all LP group procedures, whereas the other (K.S.K.) performed all UL procedures throughout the study period. The choice of surgical technique was based on individual surgeon preference. This study was approved by the Institutional Review Board (IRB) of our hospital. Informed consent was waived due to the retrospective nature of the study.

### 2.2. Surgical Techniques

**Figure 1:**
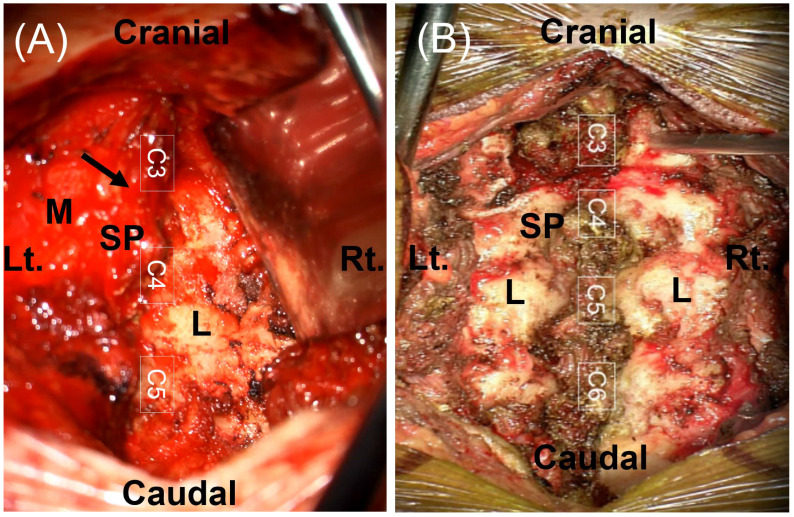
Comparison of surgical exposure following posterior muscle dissection. (**A**) Unilateral Hemilaminectomy (UL): The paraspinal muscles are retracted unilaterally, exposing only the ipsilateral lamina (L) and the base of the spinous process (SP). The contralateral musculature and the supraspinous ligament remain preserved, maintaining the structural continuity of the posterior tension band (arrow). (**B**) Laminotomy with laminoplasty (LP): Bilateral subperiosteal dissection is performed to expose the spinous process (SP) and laminae (L) on both sides. The posterior ligamentous complex is fully exposed. (SP, spinous process; L, lamina; M, paraspinal muscle). Note that the surgical levels shown are C3–C5 in (**A**) and C3–C6 in (**B**).

**Figure 2:**
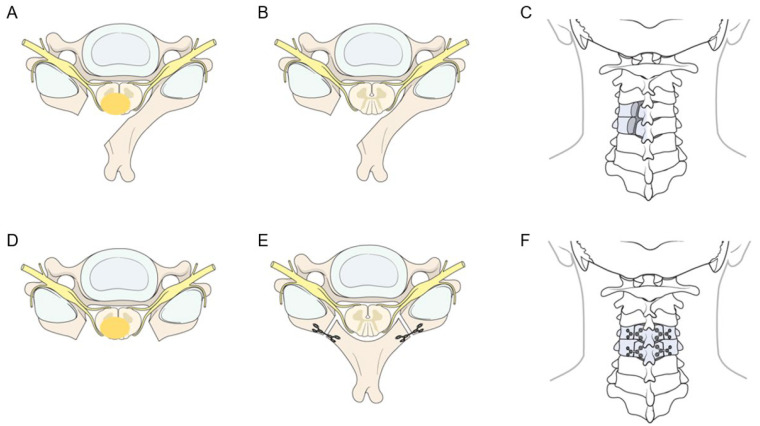
Schematic illustration of surgical procedures for cervical IDEM tumors. (**A**–**C**) Unilateral hemilaminectomy (UL) group: (**A**) Axial view showing unilateral lamina removal and exposure of the tumor mass (yellow ellipse). (**B**) Axial view following complete tumor resection, with the contralateral lamina remaining intact. (**C**) Posterior (coronal) view in the prone position after tumor removal, demonstrating the preservation of the contralateral structures and midline ligamentous complex. (**D**–**F**) Laminotomy with laminoplasty (LP) group: (**D**) Axial view demonstrating bilateral lamina removal for tumor exposure; the tumor mass is indicated by a yellow ellipse. (**E**) Axial view after anatomical reconstruction of the laminar arch using a titanium mini-plate system. (**F**) Posterior (coronal) view in the prone position showing the reconstructed laminar arch.

UL was performed based on previously established minimally invasive protocols [12]. Following the induction of general anesthesia, patients were positioned prone with continuous intraoperative neurophysiological monitoring, including somatosensory-evoked potential (SSEP) and motor-evoked potentials (MEPs). A small midline skin incision was made, and the paraspinal muscles were dissected subperiosteally on the symptomatic side only. This approach allowed a focused surgical corridor while meticulously preserving the midline ligamentous complex and the contralateral musculoskeletal structures (Figure 1A). Under microscopic visualization, a high-speed drill was used to perform the UL. Meticulous care was taken to preserve the base of the spinous process to maintain structural continuity with the contralateral lamina. To ensure postoperative stability, iatrogenic facet joint violation was strictly limited, generally aiming to preserve more than 50% of the articular process, consistent with established biomechanical safety thresholds [13]. For ventral or ventrolateral tumors, the operating table was rotated to optimize the surgical trajectory and visualization, facilitating safe tumor excision without unnecessary manipulation of the spinal cord. Upon completion of GTR, the dura was closed primarily with 6-0 Prolene sutures (Ethicon, Somerville, NJ, USA) and reinforced with fibrin sealant (Tisseel; Baxter Healthcare, Deerfield, IL, USA) (Figure 2A–C).

LP with reconstruction followed a standard bilateral posterior midline approach as previously reported [14]. Following a midline incision, bilateral subperiosteal dissection was performed to elevate the paraspinal musculature and fully expose the posterior bony elements (Figure 1B). Using a high-speed drill, bilateral gutters were created at the transition between the laminae and the facet joints to facilitate en bloc resection. This technique allowed the entire posterior laminar arch to be removed as a single unit, providing a wide, comprehensive surgical corridor for microscopic tumor resection. After tumor excision and dural closure, the posterior arch was repositioned to its original anatomical site to restore the spinal canal roof. Reconstruction was rigidly secured with a titanium mini-plate system (Lorenz Plating System; Walter Lorenz Surgical, Jacksonville, FL, USA), with plates fixed to the lateral masses or the remaining bony structures using micro-screws (Figure 2D–F). This anatomical restoration provides a protective framework for the spinal cord and a stable substrate for reattachment of the paraspinal muscles, aiming to minimize the formation of post-laminectomy membranes and preserve the structural integrity of the spinal column.

The specific surgical procedures and anatomical outcomes for both techniques are schematically compared in Figure 2. The LP group highlights the process of bilateral laminar removal followed by anatomical reconstruction using a titanium mini-plate system to restore the spinal canal roof. In contrast, the UL group focuses on the unilateral surgical corridor, emphasizing the preservation of the contralateral musculoskeletal structures and the midline ligamentous complex after tumor resection. This visual comparison underscores the biomechanical rationale for the superior motion preservation observed in the UL group by maintaining the structural integrity of the posterior tension band.

### 2.3. Radiographic Evaluation

All radiographic measurements were performed using standardized digital imaging software (INFINITT PACS version 7.0; Infinite Healthcare, Seoul, Republic of Korea). To minimize observer bias, two experienced neurosurgeons, blinded to the surgical technique and clinical outcomes, independently measured all parameters. Each observer repeated measurements twice at one-month intervals, and the mean values were used for analysis. Intra- and inter-observer reliability were assessed using the intraclass correlation coefficient (ICC); all ICC values exceeded 0.8, indicating excellent reliability. GTR was defined as complete tumor removal, with no residual enhancement on magnetic resonance imaging (MRI).

Sagittal parameters were evaluated preoperatively, at one month, and at one year postoperatively. These included the C2–C7 Cobb angle, measured between the lower endplates of C2 and C7, and the C2–C7 sagittal vertical axis (SVA), defined as the horizontal distance from the C2 plumb line to the posterior superior corner of the C7 vertebral body. The T1 slope was determined as the angle between a horizontal line and the upper endplate of the T1 body. Global cervical ROM was calculated as the difference in the C2–C7 Cobb angle between maximal flexion and extension lateral radiographs. Segmental ROM was defined as the change in the Cobb angle of the involved motion segment(s) during dynamic flexion-extension. To ensure analytical consistency, the operative level (the number of involved vertebral levels in Table 1) was defined as the total number of laminae resected for surgical exposure. In contrast, in the subsequent subgroup analysis, patients were stratified by clinical tumor extent (single level vs. multi-level) as determined by preoperative MRI.

**Figure 3:**
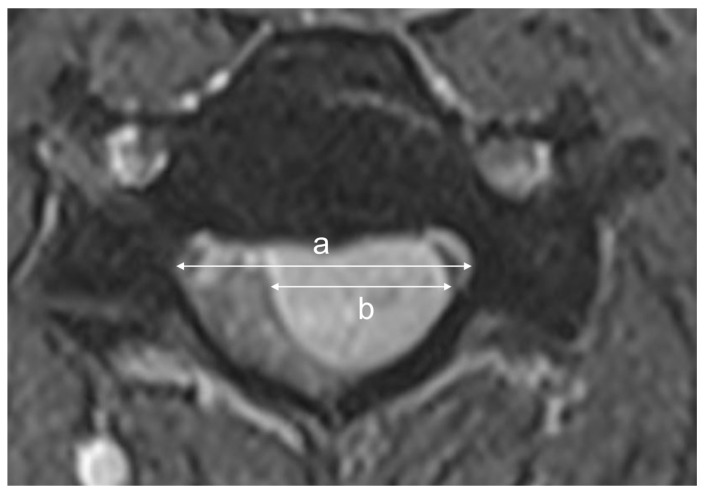
Measurement method for the Tumor-Canal Invasion Ratio (TCR). On the axial magnetic resonance imaging (MRI) slice showing the maximal tumor dimension, the spinal canal width (a) is defined as the interpedicular distance. The maximal tumor width (b) is measured at the same level. The TCR is calculated using the formula: TCR = (b/a) × 100 (%).

**Figure 4:**
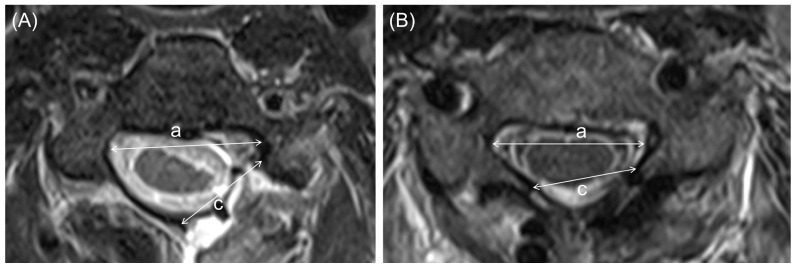
Measurement method for the Dura Exposure Ratio (DER). The extent of surgical exposure is quantified on postoperative axial images. (**A**) In the Unilateral Hemilaminectomy (UL) group, the bony opening (c) is limited to the ipsilateral side, preserving the contralateral lamina. (**B**) In the Laminotomy with laminoplasty (LP) group, the exposure extends bilaterally. The DER is calculated as the ratio of the width of the bony opening (c) to the spinal canal width (a): DER = (c/a) × 100 (%).

The surgical corridor and tumor burden were quantified using the tumor–canal invasion ratio (TCR; Figure 3) and the dura exposure ratio (DER; Figure 4) [15]. To objectively evaluate the adequacy of the surgical workspace relative to the tumor burden, we employed these ratios, as the extent of dural exposure is inherently dictated by the surgical technique. The TCR was calculated as the percentage of the maximal tumor width relative to the spinal canal width, and the DER was defined as the width of the surgical dural exposure or bony opening relative to the spinal canal width. For these calculations, the spinal canal width was measured as the interpedicular distance at the corresponding level on axial MRI.

Facet joint integrity was assessed on postoperative axial imaging and graded according to a 4-point scale based on the biomechanical thresholds established by Zdeblick et al. [13]. Grade 1 was defined as 100% intact (no bony resection), Grade 2 as less than 50% violation (at least 50% of the joint preserved), Grade 3 as 50% or more violation (less than 50% preserved), and Grade 4 as total resection or complete destruction of the joint. For the UL group, only the ipsilateral facet joint was graded. In the LP group, both facet joints were evaluated, and the higher-grade facet joint was recorded for analysis.

### 2.4. Statistical Analysis

The normality of data distribution was assessed using the Shapiro–Wilk test. Continuous variables were expressed as mean ± standard deviation (SD). For comparisons between the UL and LP groups, the independent *t*-test (including Welch’s *t*-test to account for unequal variances and small sample sizes) was used for normally distributed data, while the Mann–Whitney U test was applied for variables that did not follow a normal distribution. Categorical variables were compared using the chi-square test or Fisher’s exact test, as appropriate. To minimize potential selection bias inherent to this retrospective comparison, propensity score matching (PSM) was performed. The propensity score was estimated using multivariable logistic regression with surgical approach (UL vs. LP) as the dependent variable. Clinically relevant pre-treatment covariates were included in the propensity model, including age, sex, BMI, tumor location, pathology type, and baseline radiologic alignment parameters (ROM, C2–7 Cobb angle, C2–7 SVA, T1 slope, and segmental ROM). Patients were matched in a 1:1 ratio using nearest-neighbor matching without replacement based on the logit of the propensity score. In the matched cohort, continuous variables were compared using paired t-tests when normally distributed or Wilcoxon signed-rank tests when normality assumptions were violated. Categorical variables were analyzed using exact McNemar tests for paired binary data. A two-sided *p*-value < 0.05 was considered statistically significant. All statistical analyses were performed using IBM SPSS Statistics for Windows, version 26.0 (IBM Corp., Armonk, NY, USA).

## 3. Results

### 3.1. Demographic and Tumor Characteristics

A total of 38 patients were included in the final analysis, including 20 patients in the UL group and 18 in the LP group. As shown in Table 1, baseline demographic factors were comparable between the two cohorts. There were no significant differences in age, sex distribution, BMI, or bone mineral density T-scores. Regarding tumor characteristics, the number of involved vertebral levels, preoperative tumor width, and TCR did not differ between groups. In all included cases, GTR was achieved, as confirmed by both immediate postoperative and one-year postoperative MRI.

### 3.2. Operative Data and Facet Joint Preservation

The UL group had shorter operative times (178.05 ± 61.89 vs. 276.06 ± 121.76 min, *p* = 0.003) and lower estimated blood loss (197.50 ± 75.18 vs. 350.00 ± 112.13 mL, *p* < 0.001) than the LP group. Although the DER was significantly lower in the UL group (61.86 ± 8.72% vs. 82.95 ± 10.64%, *p* < 0.001), GTR was achieved in all patients in both groups. Regarding facet joint integrity, the LP group maintained a 100% preservation rate (Grade 1), whereas the UL group had a 25.0% (5/20) rate of facet joint violation (Grade ≥ 2) (*p* = 0.048; Table 2).

### 3.3. Postoperative Alignment and Mobility Outcomes

**Figure 5:**
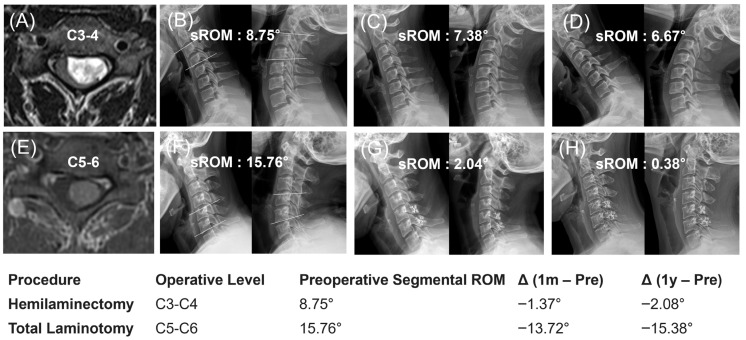
Representative cases demonstrating postoperative segmental mobility preservation. (**A**–**D**) Unilateral Hemilaminectomy (UL) at the C3–4 level (Case 1, 40-year-old female). The preoperative segmental range of motion (sROM) was 8.75° (**A**,**B**), which was well-preserved at 6.67° at 1 year postoperatively (**C**,**D**), resulting in a minimal mobility change (Δ) of −2.08°. (**E**–**H**) Laminotomy with laminoplasty (LP) at the C5–6 level (Case 2, 39-year-old female). The preoperative sROM was 15.76° (**E**,**F**), which significantly decreased to 0.38° at 1 year postoperatively (**G**,**H**), representing a profound loss of mobility (Δ: −15.38°). Note that while global sagittal alignment was maintained in both surgical approaches, the UL group demonstrated superior preservation of physiologic motion at the operated segment compared to the LP group. Abbreviations: sROM, segmental range of motion (defined as the angular difference between flexion and extension at the operated level); Δ, change.

Preoperative radiographic parameters showed no significant differences between the two groups (Table 3). Postoperatively, changes (Δ) in the C2–C7 Cobb angle, SVA, and T1 slope (−1.44 ± 10.71° in UL vs. 3.41 ± 7.44° in LP, *p* = 0.118) were not significantly different between the groups at both one month and one year. The change in global ROM at one year also showed no significant intergroup difference (−10.87 ± 17.02° for UL vs. −7.55 ± 14.03° for LP, *p =* 0.519). Notably, the decrease in segmental ROM was significantly smaller in the UL group than in the LP group at both one month (−2.11 ± 5.71° vs. −6.03 ± 5.33°, *p =* 0.048) and one year (0.06 ± 7.72° vs. −6.42 ± 8.29°, *p =* 0.017) postoperatively (Figure 5).

### 3.4. Subgroup Analysis According to the Number of Operated Levels

We further evaluated the impact of the number of surgical levels on motion preservation (Table 4). In one-level surgeries, the UL group showed a smaller reduction in segmental mobility than the LP group, although this did not reach statistical significance (−1.21 ± 6.85° vs. −4.76 ± 8.02°, *p* = 0.205). Notably, within this limited multi-level cohort (n = 8), the UL group showed significantly superior preservation of segmental ROM compared with the LP group (11.53 ± 6.68° vs. −9.73 ± 8.51°, *p* = 0.020). Although these results suggest a potential motion-sparing benefit in more extensive resections, these findings should be interpreted with caution as preliminary and exploratory due to the small sample size in this subgroup.

### 3.5. Impact of Facet Joint Violation on Stability (Subgroup Analysis)

A subgroup analysis was conducted within the UL group to evaluate the impact of iatrogenic facet violation (Table 5). The intact group (Grade 1, n = 15) showed significantly better preservation of segmental ROM at one year than the violated group (Grade 2 and 3, n = 5) (2.19 ± 5.52° vs. −6.32 ± 10.44°, *p* = 0.029). However, no significant differences were observed in the one-year postoperative change for other radiographic parameters, specifically the C2–C7 Cobb angle (*p* = 0.717), SVA (*p* = 0.904), and T1 slope (*p* = 0.207). These results suggest that while facet integrity is important for maximizing motion-sparing, the overall spinal alignment remains stable even with minor facet violation.

### 3.6. Interobserver Reliability

Inter-observer agreement for radiological measurements showed consistently high reliability across all evaluated parameters. The intraclass correlation coefficients (ICCs) ranged from 0.801 to 0.999, demonstrating good to excellent reliability for cervical sagittal alignment metrics, including the C2–C7 Cobb angle, SVA, T1 slope, global ROM, and segmental ROM (Table 6).

### 3.7. Comparison of Baseline Characteristics and Outcomes After Propensity Score Matching

To clarify the population discrepancy, the main results presented in Table 1, Table 2, Table 3 and Table 4 represent the total cohort (N = 38), while the Supplementary Tables S1 and S2 present the results of the propensity score-matched cohort (N = 36). After PSM yielded 18 matched pairs (n = 36) using 1:1 nearest-neighbor matching without replacement. Baseline demographic and preoperative radiologic characteristics in the matched cohort are presented in Supplementary Table S1. After matching, no statistically significant differences were observed between the UL and LP groups across demographic variables, including age, sex distribution, BMI, tumor location, and pathology status. Operative time, estimated blood loss, TCR, DER, and facet preservation grade were compared between groups. Differences between UL and LP remained consistent with those observed in the unmatched analysis.

Preoperative radiographic parameters, including cervical ROM, C2–7 Cobb angle, C2–7 SVA, T1 slope, and segmental ROM, were comparable between groups in the matched cohort (Supplementary Table S2). Radiographic outcomes, including changes in cervical alignment and mobility parameters at 1 month and 1 year postoperatively, showed patterns similar to those observed in the primary analysis. The direction and magnitude of group differences were preserved in the matched cohort. Overall, the matched analysis confirmed that the primary findings were not attributable to baseline demographic or radiographic imbalance.

## 4. Discussion

This study demonstrates that the UL group achieved tumor resection rates comparable to LP and maintained global sagittal alignment, while significantly preserving segmental ROM at the operated level. Although UL was associated with a higher rate of radiographic facet violation compared to the facet-sparing LP, our subgroup analysis confirmed that this did not result in radiographic evidence of instability. Notably, the UL group demonstrated significantly better preservation of segmental ROM even at the early postoperative stage (1 month) and showed near-complete recovery by one year postoperatively, suggesting its potential as a functionally advantageous motion-sparing technique. These findings are clinically significant because preserving motion and maintaining sagittal alignment are especially important in patients with intradural tumors, underscoring the critical need to balance effective tumor removal with structural preservation during surgery.

A critical concern regarding UL is the potential for iatrogenic instability due to partial facetectomy [13]. In our series, we observed no significant correlation between the occurrence of facet violation and postoperative instability parameters. This clinical stability can be explained by two biomechanical factors. First, the extent of resection generally remained within the “biomechanically safe zone.” As demonstrated by Zdeblick et al. [13], cervical facet resection of up to 50% does not result in significant segmental instability; significant decreases in torsional stiffness are typically triggered only when resection exceeds 50%. In our study, most violations (3/5) were limited to Grade 2 (<50% resection), falling strictly within this safe limit. Although no immediate instability was observed within the one-year follow-up, the significantly higher rate of facet joint violation in the UL group warrants caution regarding potential long-term implications, such as late-onset deformity or adjacent segment disease. Second, the preservation of the Posterior tension Band and the entire contralateral complex plays a pivotal compensatory role. Unlike total laminectomy, which necessitates bilateral detachment of the paraspinal musculature, UL leaves the contralateral muscle attachments, facet joint, and posterior ligamentous complex (PLC) entirely intact [13]. Our findings indicate that this “unilateral anchor” effect provides sufficient torsional stiffness to maintain sagittal alignment and prevent progressive deformity, effectively compensating for partial ipsilateral facet violation [16]. This observation further supports the biomechanical principle that preservation of the posterior column structures, even unilaterally, contributes substantially to maintaining cervical stability following tumor resection.

Historically, wide exposure was advocated for safe tumor removal [17]. However, our results support the concept that a focused surgical corridor is sufficient. In terms of surgical efficiency, the UL group demonstrated significantly reduced operative time and estimated blood loss compared to the LP group. These findings, particularly regarding the reduction in blood loss and soft tissue disruption, align with the advantages of minimally invasive approaches reported in previous studies [18,19,20]. Based on our results, UL may represent a balanced strategy that combines adequate visualization with limited structural disruption, rather than simply serving as a narrower exposure technique. Furthermore, the significantly shorter operative time in the UL group is a major clinical advantage, as reduced surgical duration is widely recognized to correlate with lower risks of surgical site infection and may facilitate postoperative recovery.

Although the UL group had a significantly lower DER (61.86%) than the LP group (82.95%), GTR rates were equivalent in both groups (100%). Our findings are consistent with the principles described by Yasargil et al. [21], who demonstrated that a unilateral partial UL provides a sufficient surgical corridor for the safe manipulation and complete removal of intradural tumors. Furthermore, our approach aligns with the biomechanical principles highlighted by Lee et al. [22], who demonstrated that sparing the posterior ligamentous complex—specifically the supraspinous and interspinous ligaments—is critical for maintaining segmental stiffness and preventing postoperative kyphosis. By utilizing the microscope’s tilting capability and high-speed drills, we optimized this “limited bony window” to achieve surgical radicality while maximizing structural preservation [23]. Unlike prior reports that primarily emphasized neurological recovery or the extent of resection, the present study also highlights longitudinal radiographic alignment and motion preservation, which may have implications for long-term spinal mechanics beyond immediate tumor control.

The most distinct advantage of UL observed in this study was the preservation of segmental mobility. In the LP group, we observed a significant reduction in segmental ROM (−6.42 ± 8.29°) despite the reconstruction of the posterior arch at 1 year. This “functional stiffening” likely results from the combined effects of extensive bilateral muscle detachment, subsequent post-surgical fibrosis, and rigid plate fixation. This phenomenon aligns with the long-term findings of Wada et al. [24] regarding cervical laminoplasty. They reported that despite preserving the lamina to avoid fusion, the range of motion decreased significantly over time (to approximately 29% of preoperative levels), often resulting in functional rigidity similar to fusion. Our subgroup analysis indicated that the UL group maintained significantly better segmental mobility in multi-level cases (*p =* 0.020; Table 4). However, given the small number of patients in this subgroup (n = 8), these data primarily represent an observable trend rather than a definitive conclusion. Although the unilateral approach may help mitigate the cumulative loss of mobility in multi-level surgeries, further studies with larger cohorts are required to confirm the robustness of this motion-sparing effect in extensive resections. Collectively, our results suggest that UL, by sparing the contralateral kinematics and paraspinal musculature, appears to minimize such rigidification and better maintain the physiologic motion of the cervical spine [22,25]. From a clinical standpoint, careful preservation of facet integrity and posterior soft tissues may be key determinants of optimizing postoperative biomechanical outcomes when employing unilateral approaches.

To address potential baseline imbalance and selection bias inherent in this retrospective design, we performed a PSM analysis. In the matched analysis, baseline demographic characteristics and preoperative cervical alignment parameters were comparable between the UL and LP groups. Importantly, the overall pattern of surgical and radiographic outcomes observed in the primary analysis was preserved in the matched cohort. The direction and magnitude of group differences remained consistent, suggesting that the principal findings were not driven by baseline heterogeneity. These results reinforce the internal validity of the present study. While complete elimination of confounding cannot be guaranteed in any observational analysis, the consistency between unmatched and matched comparisons supports the robustness of the comparative findings. Given the relatively small sample size characteristic of cervical intradural extramedullary tumors, a randomized comparison is unlikely to be feasible. Within these practical constraints, a structured propensity score framework provides a methodologically sound approach to reduce observable confounding and strengthen causal inference.

This study has several limitations. First, it is a retrospective, single-center analysis with a relatively small sample size, which may introduce selection bias and limit the generalizability of the results. Although propensity score matching was performed to reduce baseline imbalances and improve comparability between groups, residual confounding cannot be completely excluded. Second, although we primarily focused on radiological parameters, patient-reported clinical outcomes such as VAS or mJOA scores were not comprehensively assessed due to the retrospective nature of the study and incomplete medical records. However, we confirmed that no patients experienced neurological deterioration or major complications during the follow-up period. Third, although the one-year follow-up is sufficient to assess early fusion or overt instability, it may be insufficient to fully evaluate long-term sequelae, such as the development of kyphotic deformity or adjacent segment degeneration. Fourth, while we primarily focused on radiographic parameters, the association between motion preservation and patient-reported outcomes, particularly chronic axial neck pain and health-related quality of life, was not comprehensively assessed. Fifth, surgeon-related bias cannot be entirely excluded, as specific techniques were predominantly performed by two senior surgeons. Nevertheless, both surgeons are senior neurosurgeons with over 20 years of experience, ensuring a high level of technical proficiency. Future prospective studies with larger cohorts and longer follow-up periods are warranted to further validate the long-term clinical implications of this motion-sparing strategy.

## 5. Conclusions

UL provides a sufficient surgical corridor for GTR of cervical IDEM tumors, achieving oncological and radiographic outcomes comparable to LP while offering superior operative efficiency. The principal advantage of this approach appears to be the preservation of segmental mobility from the early postoperative period, with subgroup analysis suggesting that this motion-preserving effect may be maintained even in multi-level surgeries. Although a higher rate of radiographic facet violation was observed, postoperative spinal stability was not compromised in our cohort. Therefore, UL may serve as a safe and functionally advantageous alternative in appropriately selected patients with cervical IDEM tumors. Based on our findings, our institution now preferentially considers UL as a primary motion-preserving strategy, particularly for lesions that are not excessively large or strictly positioned in the ventral midline, to optimize postoperative biomechanical outcomes.

## Figures and Tables

**Table 1:** Patient demographics and tumor characteristics (Total cohort, N = 38).

Variable	UL (n = 20)	LP (n = 18)	*p*-Value
Demographics			
Age (yr)	54.00 ± 15.25	49.83 ± 17.70	0.445
Sex (female:male)	11:9	13:5	0.330
BMI (kg/m^2^)	21.71 ± 5.86	22.92 ± 3.47	0.441
BMD (T-score)	−1.02 ± 0.74	−1.59 ± 0.93	0.134
No. of operated vertebrae	2.10 ± 0.31	2.17 ± 0.86	0.747
Tumor Features			
Max tumor width (mm)	14.00 ± 4.66	15.28 ± 4.70	0.405
TCR (%)	60.25 ± 19.28	64.28 ± 17.99	0.509
Location (V/D/L)	2/2/16	2/3/13	0.816
Pathology (S/M/O)	15/3/2	9/7/2	0.223

Values are presented as mean ± standard deviation or number (%). Abbreviations: BMD, bone mineral density; BMI, body mass index; LP, laminotomy with laminoplasty; S/M/O, schwannoma/meningioma/other; TCR, tumor–canal invasion ratio; UL, unilateral hemilaminectomy; V/D/L, ventral/dorsal/lateral. Statistical significance was defined as *p* < 0.05.

**Table 2:** Operative data and surgical outcomes (Total cohort, N = 38).

Parameter	UL (n = 20)	LP (n = 18)	*p*-Value
Operation time (min)	178.05 ± 61.89	276.06 ± 121.76	0.003
Estimated blood loss (mL)	197.50 ± 75.18	350.00 ± 112.13	<0.001
DER (%)	61.86 ± 8.72	82.95 ± 10.64	<0.001
Facet Joint Integrity, n (%) ^†^			0.048
Intact (Grade 1)	15 (75.0%)	18 (100.0%)	
Violated (Grade ≥ 2)	5 (25.0%)	0 (0.0%)	
Extent of Resection, n (%)			>0.999
GTR	20 (100.0%)	18 (100.0%)	

Values are presented as mean ± standard deviation or number (%). Abbreviations: DER, dura exposure ratio; GTR, gross total resection; LP, laminotomy with laminoplasty; UL, unilateral hemilaminectomy. *p* < 0.05 indicates statistical significance. ^†^ Facet joint preservation grade was classified based on the biomechanical thresholds established by Zdeblick et al. [13]: Grade 1, 100% intact; Grade 2, <50% violation (≥50% preserved); Grade 3, ≥50% violation (<50% preserved); Grade 4, total resection.

**Table 3:** Longitudinal radiographic outcomes: alignment and mobility changes (Total cohort, N = 38).

Parameter	Time Point	UL (n = 20)	LP (n = 18)	*p*-Value
C2–C7 Cobb angle (°)	Preoperative	14.76 ± 10.50	15.45 ± 12.17	0.854
	Δ (1m–Pre)	−0.48 ± 8.05	−0.57 ± 11.02	0.979
	Δ (1y–Pre)	−0.10 ± 11.64	−0.65 ± 13.25	0.892
C2–C7 SVA (mm)	Preoperative	21.05 ± 11.92	17.66 ± 11.88	0.386
	Δ (1m–Pre)	−0.22 ± 6.85	2.99 ± 8.22	0.211
	Δ (1y–Pre)	0.07 ± 4.71	3.57 ± 7.54	0.104
T1 slope (°)	Preoperative	24.86 ± 5.30	22.02 ± 6.98	0.164
	Δ (1m–Pre)	−0.94 ± 4.15	1.54 ± 4.93	0.111
	Δ (1y–Pre)	−1.44 ± 10.71	3.41 ± 7.44	0.118
Global ROM (°)	Preoperative	45.60 ± 16.12	48.54 ± 14.08	0.554
	Δ (1m–Pre)	−10.30 ± 14.34	−10.40 ± 15.18	0.985
	Δ (1y–Pre)	−10.87 ± 17.02	−7.55 ± 14.03	0.519
Segmental ROM (°)	Preoperative	11.58 ± 7.20	15.21 ± 10.84	0.227
	Δ (1m–Pre)	−2.11 ± 5.71	−6.03 ± 5.33	0.048 *
	Δ (1y–Pre)	0.06 ± 7.72	−6.42 ± 8.29	0.017 *

Values are presented as mean ± standard deviation. Abbreviations: LP, laminotomy with laminoplasty; ROM, range of motion; SVA, sagittal vertical axis; UL, unilateral hemilaminectomy. Δ (delta) indicates the change from the preoperative baseline to each postoperative time point. * indicates a statistically significant difference (*p* < 0.05) between the two groups. Segmental ROM showed significant intergroup differences at both 1 month (*p* = 0.048) and 1 year (*p* = 0.017) postoperatively.

**Table 4:** Subgroup analysis: segmental rom changes according to the number of operated levels (Total cohort, N = 38).

Subgroup	Time Point	UL (n = 18)	LP (n = 12)	*p*-Value
1-level surgery	Preoperative	11.45 ± 7.59	12.48 ± 10.38	0.755
	Δ (1m–Pre)	−2.07 ± 5.59	−6.02 ± 6.03	0.093
	Δ (1y–Pre)	−1.21 ± 6.85	−4.76 ± 8.02	0.205
	Time point	UL (n = 2)	LP (n = 6)	*p*-value
Multi-level surgery	Preoperative	12.72 ± 1.45	20.67 ± 10.44	0.348
	Δ (1m–Pre)	−2.47 ± 9.23	−6.04 ± 3.98	0.470
	Δ (1y–Pre)	11.53 ± 6.68	−9.73 ± 8.51	0.020 *

Values are presented as mean ± standard deviation or number (%). Abbreviations: LP, laminotomy with laminoplasty; ROM, range of motion; UL, unilateral hemilaminectomy. Δ (delta) indicates the change from the preoperative baseline to each postoperative time point. *p* < 0.05 indicates statistical significance. * A significant difference was observed in the multi-level subgroup (*p* = 0.020); however, these findings should be interpreted as preliminary and exploratory due to the small sample size in this subgroup (n = 8).

**Table 5:** Comparative subgroup analysis based on facet integrity within the hemilaminectomy (UL) group (n = 20).

Parameter	Time Point	Intact (Grade 1, n = 15)	Violated (Grade 2 & 3, n = 5)	*p*-Value
Global ROM (°)	Preoperative	45.20 ± 17.13	46.78 ± 14.33	0.856
	1-year Postoperative	38.48 ± 13.59	38.97 ± 22.24	0.957
	Change (Δ)	−9.29 ± 14.51	−15.60 ± 24.53	0.487
Segmental ROM (°)	Preoperative	10.34 ± 6.47	15.27 ± 8.76	0.192
	1-year Postoperative	13.43 ± 9.13	11.20 ± 7.96	0.665
	Change (Δ)	2.19 ± 5.52	−6.32 ± 10.44	0.029 *
C2–C7 Cobb angle (°)	Preoperative	15.79 ± 11.04	11.69 ± 9.03	0.464
	1-year Postoperative	17.42 ± 10.73	12.36 ± 11.53	0.424
	Change (Δ)	0.47 ± 12.95	−1.80 ± 7.18	0.717
C2–C7 SVA (mm)	Preoperative	22.39 ± 10.33	17.00 ± 16.55	0.395
	1-year Postoperative	21.48 ± 10.63	22.68 ± 13.07	0.851
	Change (Δ)	0.14 ± 4.73	−0.19 ± 5.36	0.904
T1 slope (°)	Preoperative	24.89 ± 4.77	24.78 ± 7.32	0.967
	1-year Postoperative	27.03 ± 6.35	22.52 ± 6.06	0.224
	Change (Δ)	0.34 ± 11.33	−6.76 ± 6.94	0.207

Values are presented as mean ± standard deviation or number (%). Abbreviations: ROM, range of motion; SVA, sagittal vertical axis; UL, unilateral hemilaminectomy. Statistical significance was defined as *p* < 0.05. * indicates a statistically significant difference (*p* < 0.05). In the subgroup analysis, while facet violation was associated with a greater reduction in segmental ROM (*p* = 0.029), it did not significantly affect sagittal alignment parameters such as the C2–C7 Cobb angle (*p* = 0.717) and SVA (*p* = 0.904), suggesting that spinal stability was effectively maintained.

**Table 6:** Intra- and inter-observer reliability for radiological parameters.

Radiologic Parameter	Time Point	ICC Value	Reliability Level
C2–C7 Cobb angle	Preoperative	0.999	Excellent
	1-month f/u	0.956	Excellent
	1-year f/u	0.999	Excellent
C2–C7 SVA	Preoperative	0.990 *	Excellent
	1-month f/u	0.801	Good to Excellent
	1-year f/u	0.995	Excellent
T1 Slope	Preoperative	0.998	Excellent
	1-month f/u	0.909	Excellent
	1-year f/u	0.999	Excellent
Global ROM	Preoperative	0.993	Excellent
	1-month f/u	0.999	Excellent
	1-year f/u	0.999	Excellent
Segmental ROM	Preoperative	0.999	Excellent
	1-month f/u	0.999	Excellent
	1-year f/u	0.999	Excellent

Reliability was assessed using the intraclass correlation coefficient (ICC) based on a two-way random-effects model for absolute agreement. ICC values were interpreted as follows: >0.90, excellent; 0.75–0.90, good; 0.50–0.75, moderate; and <0.50, poor. All evaluated parameters demonstrated good to excellent reliability, confirming the robustness of the radiographic analysis. Abbreviations: f/u, follow-up; ROM, range of motion; SVA, sagittal vertical axis. * The preoperative SVA value was calculated after correcting a data entry outlier for one patient in the second observer’s dataset to ensure statistical accuracy.

## Data Availability

The data presented in this study are available on request from the corresponding author. The data are not publicly available due to privacy and ethical restrictions regarding patient medical records.

## Supplementary Materials

The following supporting information can be downloaded at: https://www.mdpi.com/article/10.3390/jcm15072672/s1, Table S1: Demographic and Perioperative Characteristics in the Propensity Score–Matched Cohort; Table S2: Radiologic Parameters in the Propensity Score–Matched Cohort.

## References

[1] Mehta A.I., Adogwa O., Karikari I.O., Thompson P., Verla T., Null U.T., Friedman A.H., Cheng J.S., Bagley C.A., Isaacs R.E. (2013). Anatomical location dictating major surgical complications for intradural extramedullary spinal tumors: A 10-year single-institutional experience. J. Neurosurg. Spine.

[2] Tumialan L.M., Theodore N., Narayanan M., Marciano F.F., Nakaji P. (2018). Anatomic Basis for Minimally Invasive Resection of Intradural Extramedullary Lesions in Thoracic Spine. World Neurosurg..

[3] Sandalcioglu I.E., Hunold A., Muller O., Bassiouni H., Stolke D., Asgari S. (2008). Spinal meningiomas: Critical review of 131 surgically treated patients. Eur. Spine J..

[4] Albert T.J., Vacarro A. (1998). Postlaminectomy kyphosis. Spine.

[5] VanPelt C., Ulibarri J.A., Kang J.D. (2006). Cervical kyphosis following laminectomy: Etiology, prevention, and surgical reconstruction. Semin. Spine Surg..

[6] Naganawa T., Miyamoto K., Hosoe H., Suzuki N., Shimizu K. (2011). Hemilaminectomy for removal of extramedullary or extradural spinal cord tumors: Medium to long-term clinical outcomes. Yonsei Med. J..

[7] Onishi F.J., Mota B., Iunes E.A., Silva C.O., Ferraro M.C., Ferreira G.B.C., Cavalheiro S. (2025). Unilateral Hemilaminectomy as Primary Treatment for Spinal Cord Tumors: Retrospective Cohort of 38 Cases with a Minimum Follow-Up of 24 Months. World Neurosurg..

[8] Chiou S.M., Eggert H.R., Laborde G., Seeger W. (1989). Microsurgical unilateral approaches for spinal tumour surgery: Eight years’ experience in 256 primary operated patients. Acta Neurochir..

[9] White A.A., Panjabi M.M. (1990). Clinical Biomechanics of the Spine.

[10] Turel M.K., D’Souza W.P., Rajshekhar V. (2015). Hemilaminectomy approach for intradural extramedullary spinal tumors: An analysis of 164 patients. Neurosurg. Focus..

[11] Pompili A., Caroli F., Crispo F., Giovannetti M., Raus L., Vidiri A., Telera S. (2016). Unilateral Laminectomy Approach for the Removal of Spinal Meningiomas and Schwannomas: Impact on Pain, Spinal Stability, and Neurologic Results. World Neurosurg..

[12] Liao D., Li D., Wang R., Xu J., Chen H. (2022). Hemilaminectomy for the removal of the spinal tumors: An analysis of 901 patients. Front. Neurol..

[13] Zdeblick T.A., Zou D., Warden K.E., McCabe R., Kunz D., Vanderby R. (1992). Cervical stability after foraminotomy. A biomechanical in vitro analysis. J. Bone Jt. Surg. Am..

[14] Kumar R., Debbarma I., Boruah T., Sareen A., Patralekh M.K., Dagar A., Kareem S.A. (2020). Flipped reposition laminoplasty for excision of intradural extramedullary tumors in the thoracolumbar spine: A case series of 14 patients. Asian Spine J..

[15] Singh P.R., Pandey T.K., Sharma R.K., Ahmad F., Kumar A., Agarwal A. (2021). Tumor Occupancy Ratio—An Imaging Characteristic Prognosticating the Surgical Outcome of Benign Intradural Extramedullary Spinal Cord Tumors. Int. J. Spine Surg..

[16] Ogden A.T., Bresnahan L., Smith J.S., Natarajan R., Fessler R.G. (2009). Biomechanical comparison of traditional and minimally invasive intradural tumor exposures using finite element analysis. Clin. Biomech..

[17] Seppala M.T., Haltia M.J., Sankila R.J., Jaaskelainen J.E., Heiskanen O. (1995). Long-term outcome after removal of spinal schwannoma: A clinicopathological study of 187 cases. J. Neurosurg..

[18] Raygor K.P., Than K.D., Chou D., Mummaneni P.V. (2015). Comparison of minimally invasive transspinous and open approaches for thoracolumbar intradural-extramedullary spinal tumors. Neurosurg. Focus..

[19] Pineiro G.T.O., Oliveira M.P.R., Sandes P.H.F., Souza D.C.R., Trocoli C., Medrado-Nunes G.S., Guirado V.M.P., Brock R.S., Quadros D.G. (2025). Hemilaminectomy vs. laminectomy for spinal tumors: A systematic review and meta-analysis. Neurosurg. Rev..

[20] Seçen A.E., Çağıl E., Divanlıoğlu D., Öcal Ö., Dalgıç A. (2024). Minimally invasive unilateral hemilaminectomy approach for the removal of spinal schwannomas impact on pain and neurological results. J. Turk. Spinal Surg..

[21] Yasargil M.G., Tranmer B.I., Adamson T.E., Roth P. (1991). Unilateral partial hemi-laminectomy for the removal of extra- and intramedullary tumours and AVMs. Adv. Tech. Stand. Neurosurg..

[22] Lee M.J., Bransford R.J., Bellabarba C., Chapman J.R., Cohen A.M., Harrington R.M., Ching R.P. (2010). The effect of bilateral laminotomy versus laminectomy on the motion and stiffness of the human lumbar spine: A biomechanical comparison. Spine.

[23] Sim J.E., Noh S.J., Song Y.J., Kim H.D. (2008). Removal of intradural-extramedullary spinal cord tumors with unilateral limited laminectomy. J. Korean Neurosurg. Soc..

[24] Wada E., Suzuki S., Kanazawa A., Matsuoka T., Miyamoto S., Yonenobu K. (2001). Subtotal corpectomy versus laminoplasty for multilevel cervical spondylotic myelopathy: A long-term follow-up study over 10 years. Spine.

[25] Tredway T.L., Santiago P., Hrubes M.R., Song J.K., Christie S.D., Fessler R.G. (2006). Minimally invasive resection of intradural-extramedullary spinal neoplasms. Neurosurgery.

