# Supplementary material for: Sagittal Alignment and Segmental Mobility After Cervical Intradural Extramedullary Tumor Surgery: A Comparative Analysis of Unilateral Hemilaminectomy and Laminotomy with Laminoplasty
Source: J Clin Med. 2026 Apr 1;15(7):2672. doi: 10.3390/jcm15072672 (PMC13073770; doi:10.3390/jcm15072672)
Supplement: Supplementary file 1 [file jcm-15-02672-s001.zip › jcm-4200372-supplementary.pdf]

Supplementary Table S1. Demographic and Perioperative Characteristics in the Propensity Score–Matched Cohort (N=36)

Variable	UL (n = 18)	LP (n = 18)	*p*-value
Age (yr)	52.17 ± 14.75	49.83 ± 17.70	0.706
BMI (kg/m²)	21.33 ± 6.02	22.92 ± 3.47	0.371
No. of operated vertebrae	2.11 ± 0.32	2.17 ± 0.86	0.086
TCR (%)	58.64 ± 19.67	64.28 ± 17.99	0.509
Operation time (min)	169.22 ± 58.19	276.06 ± 121.76	0.004
Estimated blood loss (mL)	197.22 ± 77.60	350.00 ± 112.13	< 0.001
DER (%)	61.81 ± 9.15	82.95 ± 10.64	< 0.001

Values are presented as mean ± standard deviation or number (%).
Abbreviations: BMI, body mass index; DER, dura exposure ratio; LP, laminotomy with laminoplasty; TCR, tumor–canal invasion ratio; UL, unilateral hemilaminectomy.
$p < 0.05$ indicates statistical significance.

Supplementary Table S2. Radiologic Parameters in the Propensity Score–Matched Cohort (N=36)

Parameter	Time point	UL (n = 18)	LP (n = 18)	*p*-value
C2–C7 Cobb angle (°)	Preoperative	14.95 ± 11.05	15.45 ± 12.17	0.897
	Δ (1m – Pre)	-0.46 ± 8.47	-0.57 ± 11.02	0.944
	Δ (1y – Pre)	0.44 ± 12.18	-0.65 ± 13.25	0.808
C2–C7 SVA (mm)	Preoperative	19.68 ± 11.65	17.65 ± 11.88	0.623
	Δ (1m – Pre)	-0.21 ± 7.25	2.99 ± 8.22	0.123
	Δ (1y – Pre)	-0.15 ± 4.96	3.57 ± 7.54	0.096
T1 slope (°)	Preoperative	24.10 ± 4.92	22.02 ± 6.98	0.281
	Δ (1m – Pre)	-0.43 ± 4.09	1.54 ± 4.93	0.238
	Δ (1y – Pre)	-0.64 ± 11.00	3.41 ± 7.44	0.255
Global ROM (°)	Preoperative	47.46 ± 15.33	48.54 ± 14.08	0.780
	Δ (1m – Pre)	-8.97 ± 12.65	-10.40 ± 15.18	0.455
	Δ (1y – Pre)	-11.38 ± 17.90	-7.55 ± 14.03	0.451
Segmental ROM (°)	Preoperative	12.06 ± 7.32	15.21 ± 10.84	0.375
	Δ (1m – Pre)	-2.40 ± 5.88	-6.03 ± 5.33	0.120
	Δ (1y – Pre)	-0.39 ± 7.87	-6.42 ± 8.29	0.039

Values are presented as mean ± standard deviation.
Abbreviations: LP, laminotomy with laminoplasty; ROM, range of motion; SVA, sagittal vertical axis; UL, unilateral hemilaminectomy.
Δ (delta) indicates the change from the preoperative baseline to each postoperative time point.
$p < 0.05$ indicates statistical significance.
